# Neglect of Minor Disease

**Published:** 1923-11

**Authors:** 


### NEGLECT OF MINOR DISEASE.

IN the course of his annual report upon the state of the Public Health, Sir George Newman, the Chief Medical Officer of the Ministry of Health, calls particular attention to the neglect of minor disease :—

The attention (he says) is so easily arrested by the ravages of the great diseases like smallpox, influenza, cancer, tuberculosis or venereal, that we are liable to forget that the largest burden of our invalidity, and the chief contribution to premature mortality, are due to the neglect of the comparatively minor or incipient forms of sickness and ill-health. Moreover, they are not only causes of incapacity, but they lay the foundations of the major diseases. The public health service is naturally and properly designed to combat gross evils, but it will never be really effective until and unless the origins of those gross evils are dealt with, and amongst such origins is neglected minor disease. This is one of the great spheres where the medical practitioner co-operates with the Public Health Service. It is not easy to over-estimate the harvest of sickness and death directly attributable to the neglect of such maladies as dental caries, oral sepsis, habitual constipation, neglected colds, dyspepsia, measles, or discharging ears.

### Need for Sanitary Homes.

It seems to Sir George Newman that we shut the main gates of the city against a common enemy, yet leave open a dozen doors by which he may enter in :—

We have been spending much money and labour for many years in dealing with gross disease in the adult, yet we are only now learning the old lesson that we must prevent its onset in childhood. The State and the municipality organise agencies for the health of the people, yet many of the people themselves do not live the healthy life. What is the answer to this issue ? I think it is this. We need sanitary homes, in which it is possible to live a healthy life, and millions of our people have not got such homes; we need improved medical services, and in this respect there has been extraordinary advance in the last fifteen years, but much remains to be done.

